# Coronal Repercussions of the Maxillary Central Incisor Torque in the First Set of Aligners: A Retrospective Study

**DOI:** 10.3390/dj11080186

**Published:** 2023-08-03

**Authors:** Ana Catarina Oliveira, Ana Sofia Rocha, Rita Leitão, Manuela Maia, Teresa Pinho

**Affiliations:** 1 UNIPRO—Oral Pathology and Rehabilitation Research Unit, University Institute of Health Science (IUCS), CESPU, 4585-116 Gandra, Portugal; catarina_oliveira1014@hotmail.com (A.C.O.); r.anasofia02@gmail.com (A.S.R.); ritapdaleitao@gmail.com (R.L.); 2 IPMAIA—Instituto Politécnico da Maia, Avenida Carlos de Oliveira Campos, Castêlo da Maia, 4475-690 Maia, Portugal; manuela.maia2014@outlook.com; 3 IBMC—Molecular and Cellular Biology Institute, i3S—Health Innovation and Research Institute, University of Porto, 4200-135 Porto, Portugal

**Keywords:** incisor, torque, facial biotype, clear aligners, tooth movement, Invisalign

## Abstract

Coronal torque is one of the key factors in orthodontic treatment. An adequate torque value has an impact on aesthetics and soft tissue profile. The aim of this quantitative, comparative and observational longitudinal cohort study was to analyze the efficacy of the maxillary central incisor coronal torque in the Invisalign^®^ system and evaluate the relation between coronal torque movement and patient’s facial biotype. In total, 27 patients were selected. The planned movements (TP) were obtained from the Invisalign Doctor Site^®^ using mathematical formulas that consider the T0 measurements. Pre-treatment (T0) and after full use of the first set of aligners (T1) scanners were evaluated using Geomagic^®^ Control X ^TM^ by superimposing T0 and T1 models using a transverse plane and the long axis of the tooth crown. IBM^®^ SPSS^®^ software was used for statistical purposes. We found statistically significant differences between T0 and T1 in pro-inclination and retro-inclination, as well as between achieved and planned values in pro-inclination (*p* = 0.011). We verified that hyperdivergent clinical cases presented higher mean values of coronal torque, and hypodivergent cases presented lower values. In pro-inclination, the differences between the planned and achieved values were greater in hypodivergent cases and smaller in hyperdivergent cases. In retro-inclination, the differences between the planned and achieved values were greater in normodivergent cases and smaller in hypodivergent cases. This study highlights that inefficacy is more accentuated in pro-inclination. Aligners are an effective tool for producing coronal repercussions of torque movement, being more effective in retro-inclination.

## 1. Introduction

The Invisalign^®^ system, first introduced in the 1990s by Align Technology, Inc., is now one of the most widely used orthodontic systems in the world [1]. It consists of a set of removable polyurethane aligners, made with CAD/CAM technology, developed for weekly or biweekly changes [2,3]. Each aligner must be worn between 20 and 22 h a day to increase its efficacy [4,5].

The aesthetic and comfort advantages, as well as the ease of cleaning, are points in favor of aesthetic aligners when compared to conventional fixed orthodontic appliances [6,7,8,9,10,11,12]. Disadvantages related to the use of clear aligners include limited control root movement, limited intermaxillary correction, little or no control by the operator and full-time dependence by the patient, with the appliance being removed only for eating and sanitation [13].

According to Tepedino et al., to achieve an efficient movement with the aesthetic aligner technique, it is necessary to consider the correct shape and position of the attachment, the type of material and thickness of the aligner, the amount of activation in each aligner and the sequence of movements and the associated auxiliary techniques. Also, inherent patient characteristics such as bone density, as well as crown and root morphology, can affect the behavior of teeth treated orthodontically with the clear aligner system [11].

Torque is one of the most important factors in orthodontic treatment. According to Andrews, torque represents the third key of occlusion, described as tooth movement around the midpoint in a buccolingual direction, so that the crown and root move in opposite directions. An adequate torque value has a significant impact on smile aesthetics and, consequently, on the soft tissue profile [2].

To outline a successful orthodontic treatment, the clinician needs to know the appropriate therapeutic approaches considering a good diagnosis. For this reason, an understanding of the mechanics of orthodontic movement with clear aligners must be defined right from the start to properly plan the cases with this technique, leading to more predictable treatment results.

The aim of this quantitative, comparative and observational longitudinal cohort study was to analyze the predictability and the efficacy of the maxillary central incisor coronal torque with clear aligners and evaluate the relation between coronal torque movement and patient’s facial biotype.

## 2. Materials and Methods 

### 2.1. Study Design

This work intended to analyze the efficacy and the predictability of coronal repercussions of the maxillary central incisor torque resulting from the first series of orthodontic treatment with clear aligners. Thus, two intraoral scanners were carried out at different times: one before the start of treatment (T0) and another at the end of the first series of aligners (T1). This study also intended to analyze the impact of facial biotype. 

### 2.2. Materials: Participants of the Clinical Study and Selection Criteria

The sample of this clinical trial was treated with clear aligners in a private clinic in the North region of Portugal, by a specialist in orthodontics and Invisalign^®^ Diamond Provider (T.P.). Patients’ compliance was verbally confirmed at each appointment.

To be included in this study, participants had to meet the following conditions:

Inclusion criteria:

- Individuals with definitive dentition undergoing orthodontic treatment with clear aligners;
- Individuals who had already completed, in full, the first series of orthodontic treatment with aesthetic aligners without misfits;
- Individuals with available and complete cephalometric analysis;
- Patients who presented, in the programmed table of movements taken from the Invisalign Doctor Site^®^, coronal inclination movements of maxillary central incisors above 5°.

Exclusion criteria:

- Individuals who had temporary teeth;
- Individuals whose records were incomplete;
- Individuals with cognitive or neurological disorders, with identified syndromes, history of trauma and/or tumors in the head and neck and metabolic diseases that affect the joints and/or muscles;
- Individuals who were being treated with anti-inflammatory drugs, analgesics or psychiatric medication.

The instruments used for data collection were:

- Intra-oral scanner (Itero^®^ Element 5D Plus, Align Technology, Tempe, AZ, USA);
- Itero^®^ software version 1.34.0.3 (Align Technology, Tempe, AZ, USA);
- Tables and Clincheck Pro^®^ 6.0 software (AlignTech, Santa Monica, CA, USA);
- Orthodontic and clinical patient reports;
- Geomagic^®^ Control X ^TM^ version 2022.1.0 (OQTON, X3D).

The instruments used for data processing were:

- Microsoft Excel version 16.75.2;
- IBM^®^ SPSS^®^ version 29.0.

### 2.3. Ethical Principals

This research is part of a project that was submitted for analysis and approval by the Ethics Committee of the University Institute of Health Sciences—CESPU, reference 2/CE-IUCS/2023.

### 2.4. Methods

**Figure 1:**
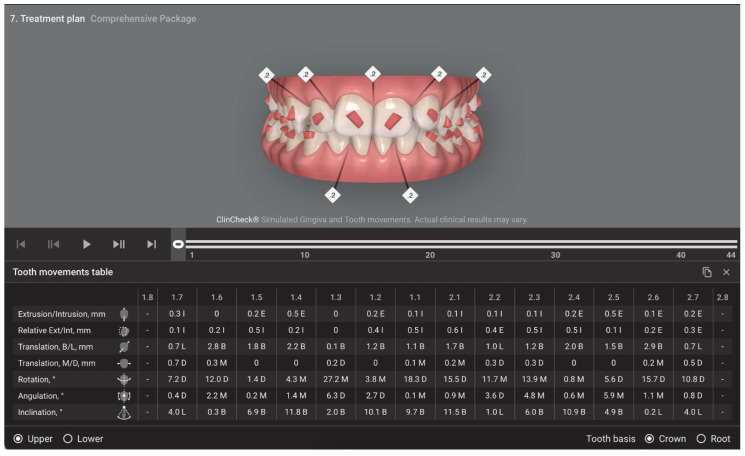
Tooth movement table on the platform from the Invisalign Doctor Site^®^ (AlignTech, Santa Monica, CA, USA).

First, the study involved collecting and evaluating the demographic and clinical characteristics of 27 participants by analyzing the clinical records. The gender and the age of the patients at the beginning of orthodontic treatment with clear aligners were collected. Through the cephalometric tracing, it was possible to analyze the overjet, overbite and facial biotype (FMA). The inclination values (°) were collected from the tooth movement tables on the Invisalign^®^ platform (Figure 1).

**Figure 2:**
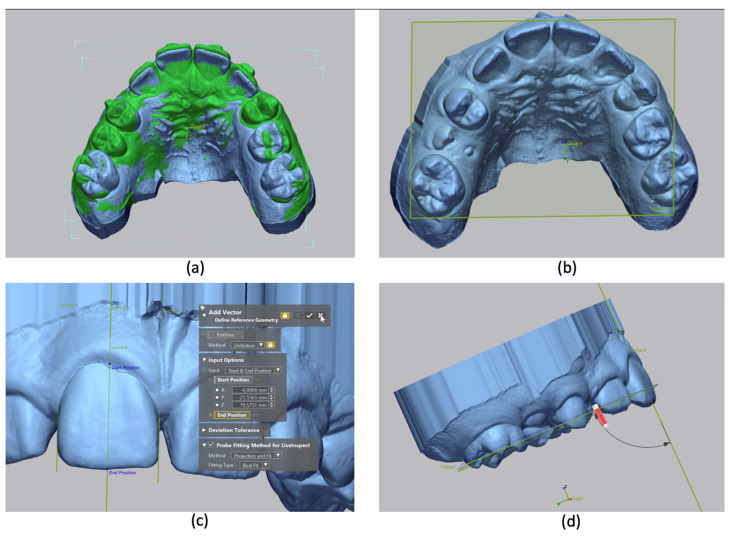
Coronal repercussions of the torque measurements on Geomagic^®^ Control X ^TM^ platform: (**a**) Superimposition of pre- and post-treatment STL models; (**b**) transverse plane generation; (**c**) virtual long axis of teeth generation; (**d**) coronal torque measurement.

To evaluate the pre- and post-treatment coronal inclination value, intraoral scanners performed at the beginning of treatment (T0) and at the end of the first series of aligners (T1) were analyzed using the Geomagic^®^ Control X ^TM^ software. To obtain the initial and the final torque value of the first series of aligners: (I) We performed the superimposition of pre- and post-treatment STL models using seven reference points in the palatal rugae. (II) Using the flood-selection tool, after segmenting the right central upper incisor and first molars, we generated a transverse plane in the initial STL (T0), common to pre- and post-treatment with reference in the first right and left molars and in the upper right central incisor. (III) After generating the transverse plane, the virtual long axis of the teeth (in pre- and post-treatment) was created, producing the connection between the most occlusal and gingival points along the midline of the buccal surface. (IV) After that, it was possible to evaluate the angle corresponding to the coronal torque of the upper central incisor. The angle between the transverse plane and the virtual long axis of the tooth corresponded to the coronal torque measurement (Figure 2).

The planned value of the patients’ coronal torque was mathematically obtained using the following formulas:

- For pro-inclination ⇒ TP = T0 + Tooth movement planned value on platform.
- For retro-inclination ⇒ TP = T0 − Tooth movement planned value on platform.

The measurement method was chosen to minimize errors, leading to greater reliability of the results. In all STL models, it was possible to make the transverse plane and the long axis of the crown of the tooth in the same way, avoiding incorrect measurements.

### 2.5. Statistical Analysis

Data analysis was performed using the IBM^®^ SPSS^®^ program (Statistical Program for Social Sciences), version 29.0 for MacOS. The level of statistical significance was set at 0.05. Descriptive statistics were performed to estimate frequencies, means, median, standard deviation, minimum and maximum. The Kolmogorov–Smirnov test was used to assess sample normality. The normality of the data led us to adopt the Wilcoxon Signed Test to compare the coronal repercussions of the torque value of the maxillary central incisor and the achieved value after completion of the first series of aligners. The same test was used to compare the programmed and achieved values at T1. To study the influence of different facial biotypes on T0 and T1 and between planned and achieved values, the Kruskal–Wallis test correction was used. The significance level used was 0.05. To determine the intraclass correlation coefficient, measurements were repeated by a second clinician (R.L.). Inter-examiner reliability was noted (ICC = 0.997).

## 3. Results

### 3.1. Characteristics of the Clinical Study Sample

**Figure 3:**
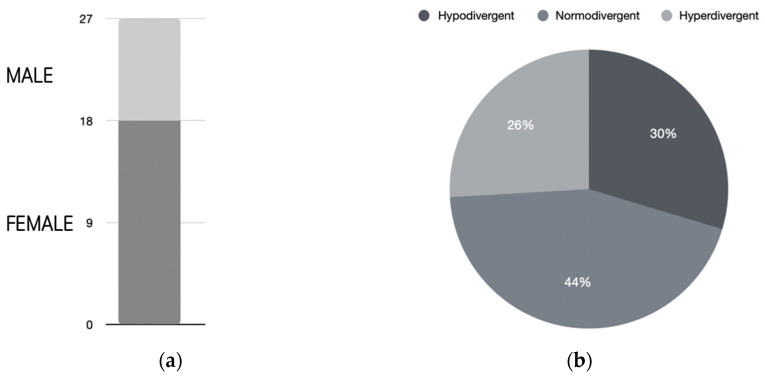
Characteristics of the clinical study sample: (**a**) gender, (**b**) facial biotype.

The sample consisted of 27 patients aged between 11 and 45 years old (mean = 25.2; SD = 11.5), of which 9 (33.3%) were male and 18 were female (66.6%) (Figure 3a). Regarding facial biotype, 8 (29.63%) were hypodivergent, 12 (44.4%) were normodivergent and 7 (25.93%) were hyperdivergent (Figure 3b).

The clinical study of the sample revealed that majority had normal overjet (77.8%; n = 21) and increased overbite (59.3%; n = 16) (Table 1).

Case complexity levels were based on clinical records using the Invisalign Doctor Site^®^ online platform. This tool classifies cases as simple, moderate or complex based on Align^®^ recommendations. In this sample, we found 19 moderate cases (70%) and 8 complex cases (30%) (Table 2).

Regarding the number of aligners used for patients in the first phase, we verified a minimum value of 24 aligners and a maximum of 55 (median = 34 aligners).

### 3.2. Efficacy and Predictability of Coronal Repercussions of Maxillary Central Incisor Torque

Table 3 summarizes the differences between the coronal torque values of the maxillary central incisor before and after the first series of aligners. For each sub-sample (pro-inclination and retro-inclination), there were statistically significant differences in terms of the coronal torque movement between T0 and the achieved T1 on the upper central incisor. Considering the pro-inclination sample, the average value of the initial coronal torque movement was less than the average coronal torque value achieved (Z = −5.571; *p* ≤ 0.001). Considering the retro-inclination sample, the average value of the coronal initial torque movement was higher than the average coronal torque value achieved (Z = −2.903; *p* = 0.004).

Table 4 summarizes the differences between the achieved and programmed coronal torque values of the maxillary central incisor after the first series of aligners. Considering the part of the sample who needed pro-inclination, we verified the existence of statistically significant differences (Z = −2.707; *p* = 0.007). Considering the part of the sample who needed retro-inclination, we verified no statistically significant differences (Z= −1.295; *p* = 0.195). Considering the pro-inclination sample, the average coronal value of the programmed torque movement was greater than the average coronal torque value achieved. Considering the retro-inclination sample, the average coronal value of the programmed torque movement was less than the coronal average torque value achieved.

### 3.3. Influence of Different Facial Biotypes on Coronal Repercussions of the Maxillary Central Incisor Torque

**Figure 4:**
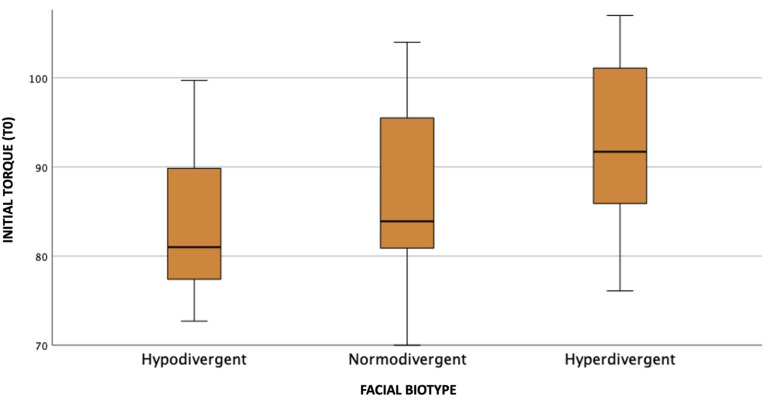
Distribution of coronal torque values according to facial biotype in T0.

**Figure 5:**
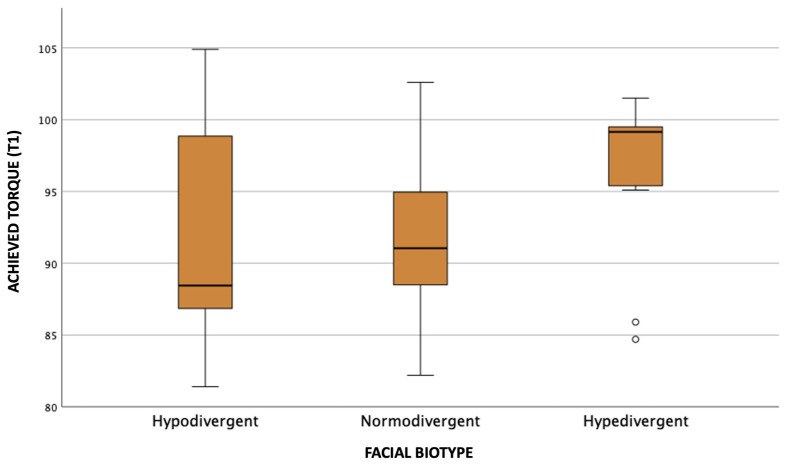
Distribution of coronal torque values according to facial biotype in T1.

Observing the graphs referring to the initial and final coronal torque movement (Figure 4 and Figure 5), it is possible to notice that individuals with the hyperdivergent biotype presented the highest value of the median coronal torque, followed by the normodivergent and hypodivergent biotypes.

**Figure 6:**
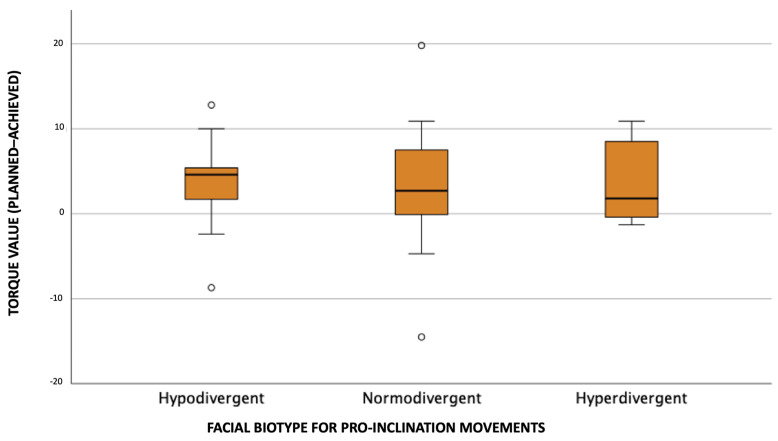
Distribution of coronal torque values (planned–achieved) according to facial biotype for pro-inclination movements.

Figure 6 allows us to verify that, in terms of maxillary central incisor coronal torque (planned–achieved) for pro-inclination movements, hypodivergent individuals had the highest median value, followed by normodivergent and hyperdivergent individuals, respectively. We verified the presence of coronal torque overexpression (negative values) in the three types of facial biotype, being more noticeable in normodivergent biotype.

**Figure 7:**
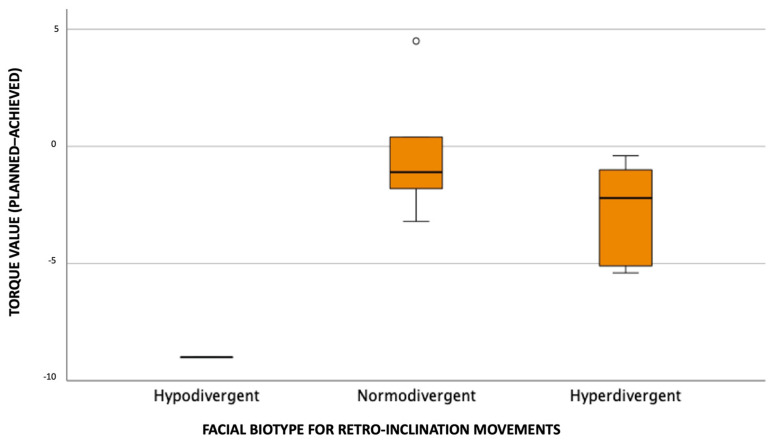
Distribution of coronal torque values (planned–achieved) according to facial biotype for retro-inclination movements.

Figure 7 allows us to verify that, in terms of maxillary central incisor coronal torque (planned–achieved) for retro-inclination movements, normodivergent individuals have the highest median value, followed by hyperdivergent and hypodivergent, respectively. We verified the presence of coronal torque overexpression (positive values) in the normodivergent biotype.

## 4. Discussion

Since the introduction of clear aligners in the dental market, the reliability of 3D digital planning has always been a subject of debate. Clear aligners have been appreciated by patients for their comfort and low aesthetic impact [7].

In addition to the mechanical force system, other factors can affect the expression of incisor torque, like rotations correction, because it will not always occur purely along the long axis of the teeth. Moreover, pre-existing spacing or crowding that requires a transverse contraction or expansion can lead to a corresponding lingually or labially directed force affecting torque expression. Also, the disparity resulting from an underestimation of the mesiodistal width of the teeth by the ClinCheck^®^ plan may result in tighter final aligners, possibly contributing to a lingually directed force, and the thickness of the attachments on the buccal surface of the incisors may cause a force directed lingually from the lips. Furthermore, in incisors, the prescribed palatal root movement/labial crown torque will experience an extrusive component of resultant force, which, together with the flexible nature of aligners, results in a gap between the tooth and the edge of the aligner on the palatal surface. For these reasons, in most studies, we found an underexpression of labial crown torque and a normoexpression or overexpression of lingual torque [2].

Inferential statistics of our study showed that the general sample and the labial crown torque group were significantly different (*p* < 0.05) in achieving the prescribed level of torque. Our reported results on the efficacy of the coronal torque movement of the maxillary central incisor corroborate previously published studies [1,2,14,15]. Other studies contradicted the results of our study, demonstrating that there were no statistically significant differences between the planned and achieved values [11,16].

This clinical study is in line with previous studies by Grunheid et al. These authors used a similar sample of patients; however, they did not subdivide the sample into retro-inclination and pro-inclination [1]. Fan-Fan Dai et al. reached the same conclusion with a sample of 30 patients; however, the aforementioned study involved cases of extraction of premolars, contrary to our study, which did not involve extraction cases [17]. Bowman et al., with a sample of 33 patients, obtained similar conclusions only including adults (18 or more years) [18]. The study of Gaddam et al. was the only one who carried out the sample division of retro-inclination and pro-inclination, obtaining results like in our study [2].

As in the present study, Gaddam et al. concluded that the difference between the predicted and achieved torque was statistically significant in the group corresponding to buccal crown (positive torque) torque for the maxillary central incisor. The subgroup corresponding to the lingual/palatal crown (negative torque) torque did not show a statistically significant torque differential (*p* > 0.05) [2].

In our study, we included planned movement values above 5°, contrary to Simon et al., who only used maxillary central incisor torque values above 10° [18]. Tepedino et al. also found no significant difference in torque differential between the predicted and achieved change using another brand of clear aligners, and the authors only evaluated the first phase of treatment made on 12 aligners [11]. In contrast, our study used a minimum of 24 aligners and a maximum of 55 aligners, with a median of 34 aligners.

Using Geomagic^®^ Control X ^TM^ software, in our clinical trial, in contrast to Gaddam et al. and Bownam et al., the superimpositions could be conducted on stable reference planes/areas (palatal rugae). So, in our study, the superimpositions of the pre- and post-treatment models were carried out using seven points located in the palatal rugae [2,14].

The transversal plane used in the present study was partially reproduced from the studies by Gaddam et al., Tepedino et al. and Bowman et al., but with some differences. Namely, the isolation of the first molars was performed instead of the most distal molars since they present greater stability. In addition, the age of the total sample, which included young people, varied between 11 and 45 years [2,11,19].

The long axis of the tooth was not traced like in the studies of Gaddam et al. and Bowman et al., considering that the margin of error between the real and virtual long axis could vary between 2° and 37.6° [2,19]. In our study, the long axis was drawn considering the reference points described by Fan-Fan Dai et al. and Catroflorio et al.—most incisal and gingival points of the midline of the buccal surface of the tooth [3,15].

Considering our results, in the labial crown torque subgroup, the mean differential torque (predicted-achieved) was 5.4°; in contrast, the mean torque differential in the lingual/palatal crown torque group was lower, corresponding to −2.4°, which showed that lingual crown torque was more effective than labial crown torque, as in Gaddam’s study [2].

The sample studied includes cases with a moderate to complex degree of complexity according to the Invisalign^®^ tool, but without extraction. In more severe cases, which need extractions, factors such as the correction of increased rotations, spacing or severe crowding can lead to changes in orthodontic forces and prevent planned movements from being carried out [2].

Chisari J. et al. found a relationship between age, sex, root length, bone levels and bone quality on orthodontic tooth movement [19].

In our selected clinical cases, we did not have power ridges. However, Castroflorio et al., who did not study the facial biotype, concluded that it is possible that aligners with power ridges may provide better control of the upper incisors than can be achieved with a preadjusted system, at least in some prescriptions [16].

The facial biotype is very useful and, like our study, describes that the excessive vertical lower facial height seen in hyperdivergent individuals leads to elongated faces, open bite and proclination of the upper central incisors. In hypodivergent individuals, the opposite happens, which can lead to situations of crossbite and retroclination of the upper central incisors [15].

In our study, we verified that, for the pro-inclination movement, the average of the differences between the planned and achieved values was greater in the hypodivergent cases and smaller in the hyperdivergent cases. On the other hand, in retro-inclination movements, the average of the differences between the planned and achieved values was greater in normodivergent cases and smaller in hypodivergent cases.

Hypodivergent individuals, who have a smaller vertical facial pattern, may generally exhibit less resistance to incisor inclination movements due to their skeletal and soft tissue characteristics. The reduced vertical facial height in hypodivergent individuals may provide a more favorable biomechanical environment for achieving incisor torque movements with less difficulty. On the other hand, hyperdivergent individuals, who have a larger vertical facial pattern, often present unique challenges in orthodontic treatment [15].

Recent studies have demonstrated that the incorporation of CBCT in the intraoral scanner by dental superimposition based on stable anatomical landmarks is an asset in the planning of orthodontic treatments [20]. The use of 3D with the incorporation of CBCT, in future studies, will allow for the assessment of the coronal repercussions of the torque movement, changes in the root and whether the planned root movements were fully achieved.

The selection bias associated with a retrospective study design was kept to a minimum by comparing the pre-treatment digital models to the end of the initial aligner sequence without misfits and with a planned torque value above 5°. This was done to evaluate dental movements classified as unpredictable, which provide a limitation on sample size; however, 54 maxillary central incisors were considered.

## 5. Conclusions

Aligners are an effective tool for producing coronal repercussions of torque movement, being more effective in retro-inclination movements.

Hyperdivergents showed more pro-inclination of the upper central incisors. In hypodivergent individuals, a retro-inclination of the upper central incisors was observed.

For the pro-inclination movement, the average of the differences between the planned and achieved values was greater in the hypodivergent cases and smaller in the hyperdivergent cases.

For retroclination, the average of the differences was greater in normodivergent cases and smaller in hypodivergent cases.

Taking these results into account, multiple treatment phases with additional aligners may be required to achieve torque goals. In addition, regular clinical control of the patient is also essential for the orthodontist to be able to assess whether the treatment is proceeding as planned.

## Figures and Tables

**Table 1:** Characteristics of the clinical study sample (overjet and overbite).

Variable	Definition	Frequency (Number)	Percentage (%)
Overjet	Decreased (<0 mm)	2	7.4
	Normal (2.5 ± 2.5 mm)	21	77.8
	Increased (>5 mm)	4	14.8
Overbite	Decreased (<0.5 mm)	2	7.4
	Normal (2.5 ± 2 mm)	9	33.3
	Increased (>4.5 mm)	16	59.3

**Table 2:** Characteristics of the clinical study sample (complexity of the case by Invisalign Doctor Site^®^).

Variable	Definition	Frequency (Number)	Percentage (%)
Complexity of the case	Simple	0	0
	Moderate	19	70
	Complex	8	30

**Table 3:** Comparison between coronal torque values of the maxillary central incisors before and after completion of the first series of aligners (Wilcoxon Signed Test).

Sample	Moment	Mean ± SD	z	*p*-Value
Proinclination	T0	83.6 ± 7.3	−5.571	<0.001 *
	T1	91.9 ± 6.4		
Retroinclination	T0	101.0 ± 3.9	−2.903	0.004 *
	T1	96.4 ± 3.6		

* Statistically significant (*p* < 0.05).

**Table 4:** Comparison between coronal torque values of the maxillary central incisors achieved and programmed after completion of the first series of aligners (Wilcoxon Signed Test).

Sample	Moment	Mean ± SD	z	*p*-Value
Pro-inclination	T1	91.9 ± 6.4	−2.707	0.007 *
	TP	95.4 ± 5.3		
Retro-inclination	T1	96.4 ± 3.6	−1.295	0.195
	TP	94.3 ± 3.8		

* Statistically significant (*p* < 0.05).

## Data Availability

Data that support this study’s findings are available from the corresponding author upon request.
